# Intermolecular Interactions Enhance the Light Absorption of a Methoxyphenol Constituent of Biomass Burning Emissions

**DOI:** 10.1021/acsestair.4c00294

**Published:** 2025-02-27

**Authors:** Colton T. Calvert, Nathan J. Huskins, Elijah G. Schnitzler

**Affiliations:** Department of Chemistry, Oklahoma State University, Stillwater, Oklahoma 74078, United States

**Keywords:** biomass burning, brown carbon, aerosol absorption, charge-transfer complexes, benzoquinone, naphthoquinone

## Abstract

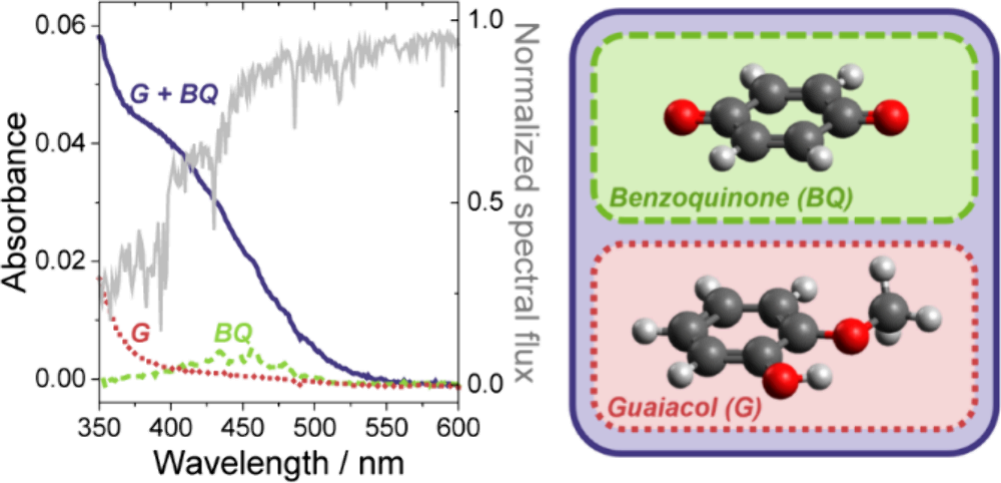

Brown carbon (BrC) components of biomass burning organic aerosol (BBOA) absorb sunlight at visible wavelengths. However, it is not clear whether the total light absorption of this BrC is simply the sum of the contributions of the individual components or whether the components can bind noncovalently to give additional absorption through charge transfer. Here, intermolecular interactions between guaiacol and quinones (1,4-benzoquinone and 1,4-naphthoquinone) were identified in proxies of the nonpolar, water-insoluble phase of BBOA, using UV–vis spectroscopy. Guaiacol and its derivatives are some of the most abundant emissions of smoldering coniferous species. Enhanced light absorption occurred instantaneously upon mixing colorless guaiacol with either quinone in *n*-heptane and did not increase with time, in contrast to the absorbance changes that would be expected for a covalent product. This enhancement decreased by about 25% as the temperature increased from 303 to 323 K, consistent with exothermic association to give complexes, yielding enthalpies of complexation of −13.3 ± 0.6 and −12.3 ± 0.4 kJ mol^–1^ for guaiacol with benzoquinone and naphthoquinone, respectively. Enhancement was also observed upon gas–liquid partitioning of benzoquinone into thin films of guaiacol, for example, with a thickness of 20 μm. This multiphase processing, mimicking partitioning of quinones into liquid BBOA, produced absorption comparable to moderately absorbing BrC from other sources, suggestive of the atmospheric relevance of these interactions.

## Abstract 2

Colorless guaiacol absorbs visible light when complexed with benzoquinone and naphthoquinone, so it is a potential component of brown carbon in biomass burning emissions.

## Introduction

Biomass burning organic aerosol (BBOA) includes a mass fraction that efficiently absorbs visible sunlight, called brown carbon (BrC),^1,2^ which is composed of compounds from the thermal decomposition (i.e., pyrolysis) and incomplete combustion of lignin and cellulose, ranging from phenolic species to substituted polycyclic aromatic hydrocarbons.^3,4^ The composition of BrC has been studied extensively using liquid chromatography coupled to parallel UV–vis diode array detectors and high-resolution mass spectrometers.^5−8^ The sum of absorbances attributable to individual compounds separated on the column is sometimes smaller than the total absorption of the whole mixture before separation.^5,9^ This discrepancy could be explained by enhanced absorption due to noncovalent interactions in the mixture,^10,11^ as has been proposed in the context of natural waters, where electron donor–acceptor, or charge-transfer, complexes between hydroxyl-substituted aromatics and quinones have been suggested to contribute to the light absorption of chromophoric dissolved organic matter (CDOM).^12,13^ There is some evidence that similar complexes could play a role in ambient BrC.^10,11^

In the aquatic environment (i.e., natural waters), however, there are significant discrepancies between studies of complexation.^14−16^ For example, loss of absorbance upon selective reduction of carbonyl groups in quinones, potentially disrupting intermolecular interactions, supports a significant role for complexes in CDOM,^12,15^ whereas steady absorbance upon changing solvent and temperature, factors that should affect these interactions, supports a minimal role for complexes.^14,16^ Historically, few studies of organic charge-transfer complexes in water have been reported in the physical organic chemistry literature,^17−19^ in part due to solute–solvent interactions and solubility limits.^20^ The subset of studies of complexes involving quinones in water is even smaller.^18,19^ Interestingly, in each of these, color developed in the aqueous solutions slowly, over the course of hours^18,19^ rather than instantaneously, as is typically observed for charge-transfer complexes.^21^ In solvent mixtures of isopropanol–water and ethanol–water, similarly slow coloration involving 1,4-benzoquinone has been attributed to its instability, reacting to form 1,4-hydroquinone.^22^ Reciprocally, 1,4-hydroquinone has recently been shown to autoxidize in water to 1,4-benzoquinone to give absorbance characteristic of CDOM.^23^ This intricate redox cycling of quinones also involves solar irradiation.^24^ Similarly, it is not yet resolved whether or to what extent charge-transfer complexes contribute to the total light absorption in the aqueous phase of deliquesced particles and cloud droplets; while selective reduction led to decreased absorbance for BBOA extracted into water,^10,25^ increasing temperature did not.^25^

**Figure 1:**
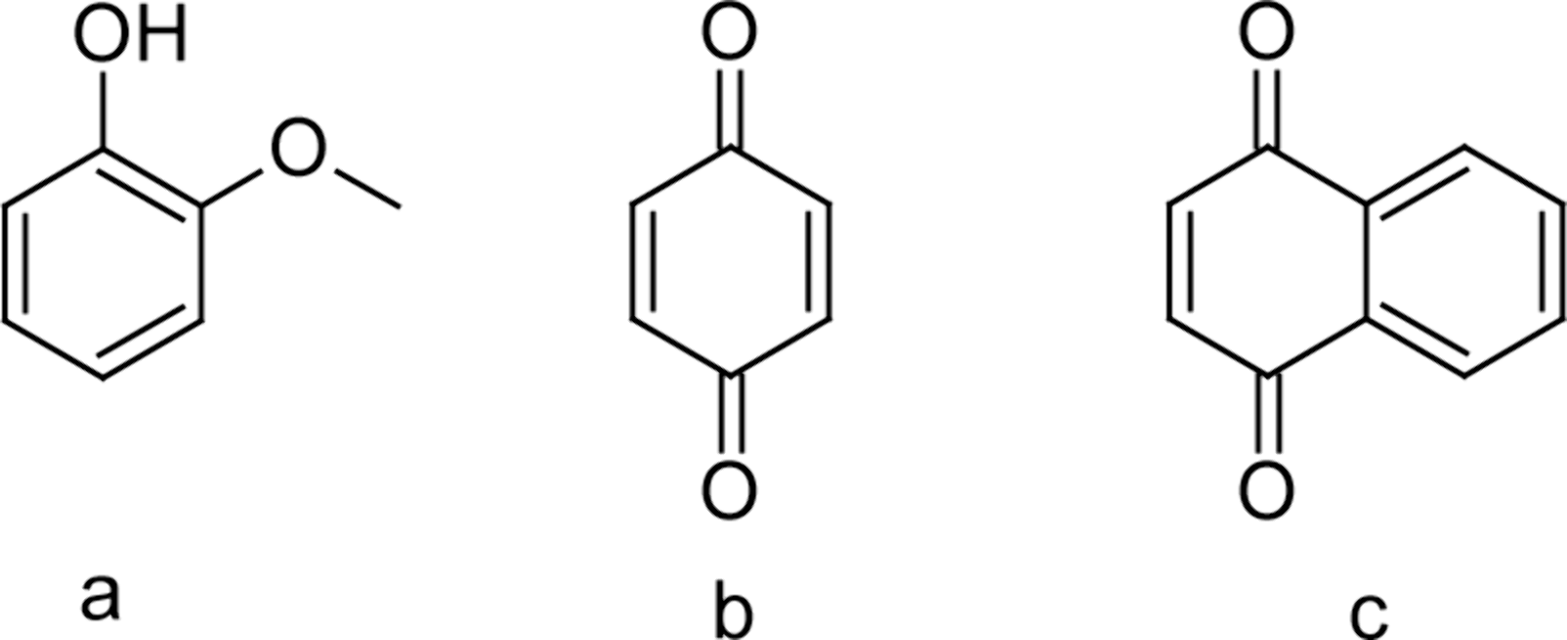
Chemical structures of (a) guaiacol; (b) 1,4-benzoquinone; and (c) 1,4-naphthoquinone.

In this work, we expressly shift this focus from the aqueous phase to the nonpolar organic phase. In the atmosphere, BBOA is composed of species with wide ranges in chemical, physical, and optical properties.^3,4^ In particular, a wide range in polarity,^26,27^ often quantified in terms of the oxygen-to-carbon ratio, can lead to liquid–liquid phase separation,^28^ with implications for multiphase processing and ice nucleation.^29,30^ The presence of a low-polarity, hydrocarbon-like organic phase in BBOA allows an additional solvent environment in which charge-transfer complexes could be playing a role in the light absorption of BrC.^31,32^ Typically, the water-insoluble fraction of BBOA is more absorbing than the water-soluble fraction,^25,27,33^ often attributed to large conjugated species, which may participate in complexes. By far, more observations of the fundamental thermodynamics of charge-transfer complexes have been made in organic solvents, including hydrocarbons, than in water.^21,34^ These systems include well-known “textbook” examples of quinhydrone-type complexes, e.g., hydroquinone complexed with benzoquinone.^35^ If similar intermolecular interactions occur in the hydrophobic organic phase of BBOA,^28,36^ they could have an important role in the light absorption and, in turn, climate effects of BrC.^37,38^ Indeed, while no temperature dependence of absorbance was observed for BBOA extracted into water (i.e., about 75% of the mass), a slight temperature dependence was observed for BBOA extracted into dimethyl sulfoxide (i.e., 100% of the mass).^25^ To date, there has been no bottom-up investigation of these complexes in BBOA to complement the top-down approaches used previously,^10,25^ necessary to provide a molecular-level understanding of which components may participate in these interactions. Here, the intermolecular interactions between guaiacol (i.e., 2-methoxylphenol, shown in Figure 1), a significant, colorless emission of biomass burning,^3,4^ and 1,4-benzoquinone and 1,4-naphthoquinone, which form in the atmosphere through combustion and oxidation,^39−41^ were investigated using UV–vis absorption spectroscopy to gain fundamental insights into complexation and absorptivity in solution as well as in thin films, which are more representative of liquid BBOA particles undergoing gas-particle partitioning.

## Materials and Methods

### Bulk Solution Experiments

Experiments were first performed in solution. Stock solutions of guaiacol (Sigma, ≥98.0%), 1,4-benzoquinone (Alfa Aesar, >98%), and 1,4-naphthoquinone (TCI, >98.0%) were prepared in *n*-heptane (Thermo, 99%). The prefixes for these compounds are omitted for the rest of our discussion. The concentration of the stock solution of benzoquinone was 1.02 × 10^–3^ M. The solubility limit was determined to be 1.11 × 10^–3^ M. Aliquots of the stock solution of benzoquinone and pure liquid guaiacol were added to a cuvette and diluted such that the concentration of benzoquinone was either 3.4 × 10^–4^ or 6.8 × 10^–4^ M and that of guaiacol was either 0.15, 0.30, 0.45, or 0.60 M. Colloidal particles of guaiacol suspended in heptane were sometimes observed upon initial mixing, so the cuvette was capped and shaken vigorously. UV–vis absorption spectra were recorded at temperatures ranging from 303 to 323 K, in increments of 5 K, to investigate any dependence on temperature. Before each spectrum was recorded, 10 min was allowed for the heating block to reach the target temperature and the solution to equilibrate at this temperature. After the target temperatures were reached, the heating block was returned to the initial temperature, and the spectrum was recorded again to determine whether any changes observed with varying temperature were reversible. These experiments were also performed with naphthoquinone in place of benzoquinone. Spectra at different temperatures were measured on a commercial dual-beam spectrometer (Varian, Cary 5000) equipped with a recirculating temperature controller. The instrument was zeroed and baseline-corrected with heptane. For selected solutions, the spectrum was also recorded in intervals of 1 h to investigate any dependence on time. After the solution was allowed to sit overnight, another spectrum was recorded at 24 h after mixing. Spectra at different times were measured on an alternate commercial dual-beam spectrometer (Varian, Cary 100). In another set of experiments, a solution of benzoquinone was added to the cuvette, and the spectrum was recorded once per hour for 3 h. At hour three, this benzoquinone solution was combined with an equivalent volume of pure liquid guaiacol, forming two immiscible layers. Every hour for the next 3 h, the layers were separated using a micropipette, and the spectrum was recorded for each before the layers were recombined to investigate liquid–liquid partitioning. After mixing, an aliquot of guaiacol from the lower layer was also analyzed using a liquid chromatograph (Agilent, 1260 Infinity) coupled to a diode array detector (Agilent, 1260 DAD HS), in order to monitor for any changes in composition with the occurrence of color (see Supporting Information).

### Multiphase Experiments

**Figure 2:**
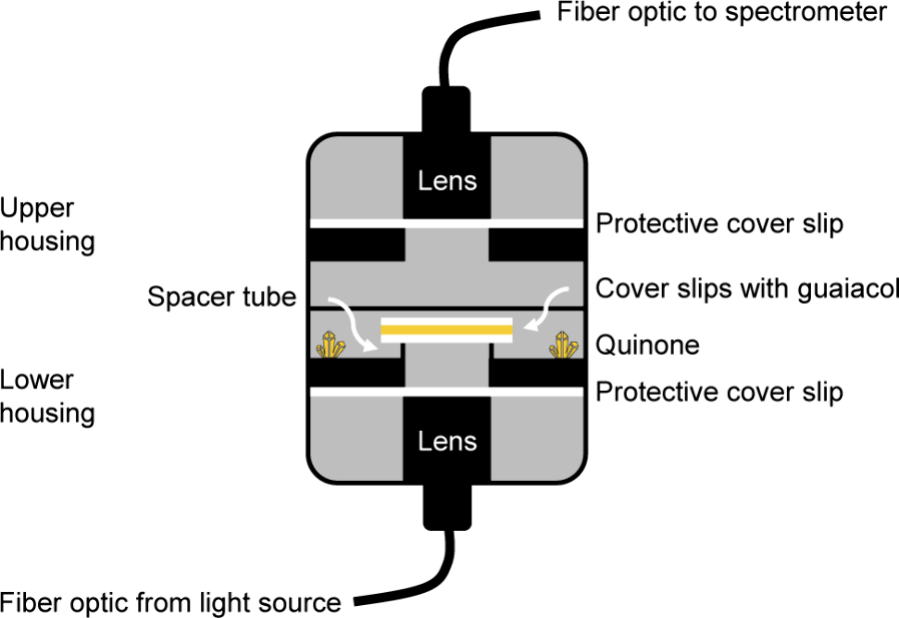
Schematic illustration of the design of the cylindrical chamber for exposure of thin films of guaiacol to gas-phase quinones and simultaneous UV–vis absorption spectroscopy.

Multiphase experiments involving liquid guaiacol and gas-phase benzoquinone or naphthoquinone were also performed in thin films. Briefly, pure liquid guaiacol was sandwiched between two circular cover glasses and exposed to benzoquinone or naphthoquinone at their respective sublimation pressures in a small cylindrical exposure chamber with a perpendicular beam of light from a broadband light source passing through the film into a spectrometer, as illustrated in Figure 2. The light source, with combined deuterium and tungsten emission (Ocean Optics, DH-2000-BAL), was connected through a 10 cm-long fiber optic (Ocean Insight, P400-010-UV-VIS) to a dispersing lens (Ocean Insight, 74-UV), the first element of the exposure chamber. The lens was housed in a 1″-long Ø1″ stackable lens tube (Thorlabs, SM1L10) using a threaded adapter (Thorlabs, AD57F). Commercially, the adapter is threaded on the outside only, so the inside was custom-threaded to receive the lens. The adapter was positioned using a retaining ring, and an O-ring was placed between the retaining ring and the adapter. To protect the lens from vapors, an 18 mm diameter circular cover glass (VWR, 48382-042) was placed between the base adapter and a Ø1″-to-Ø1/2″ optic adapter (Thorlabs, AD1), secured with another retaining ring. The optic adapter supported a 9.6 mm stainless steel spacer tube (Thorlabs, CVH100-COL), which in turn supported the two circular cover glasses sandwiching the sample. The base lens tube was secured vertically using a lens mount (Thorlabs, VG100), with the connection to the light source facing down and the lens tube threads facing up. Another O-ring was placed around the base of these threads, leaving enough room for a second lens tube (Thorlabs, model SM1L10) to be fastened to the base. In this lens tube, the reverse combination of an optic adapter (Thorlabs, AD1T), cover glass, threaded adapter (Thorlabs, AD57F), collimating lens (Ocean Insight, 74-UV), and O-ring was positioned and secured using retaining rings. These components are shown in Figure S1. The collimating lens was connected though another 10 cm-long fiber optic to a miniature spectrometer (Ocean Insight, FLAME) with a grating-based polychromator, fitted with a 25 μm aperture at the entrance. At the beginning of a multiphase experiment, either 22 mg of benzoquinone or 35 mg of naphthoquinone, both crystalline solids at room temperature, was placed in the radial groove between the base lens tube and the spacer tube. The bottom cover glass was centered on the spacer tube, a 5 μL drop of pure liquid guaiacol was dispensed onto the cover glass using a micropipette, and the top cover glass was carefully lowered onto the drop. The guaiacol was uniformly distributed across the area of the cover glasses, and the resulting film was sufficiently thin so that no guaiacol flowed over the edge. The reference and dark spectra were recorded for two cover glasses without a thin film of guaiacol. Recorded spectra were the averages of 100 individual spectra, measured in Ocean ART (Ocean Insight). Spectra were recorded every hour for up to 48 h, using a custom Python program to control Ocean ART. After each multiphase experiment, the exposure chamber was disassembled and cleaned thoroughly. In control experiments, the absorbance of guaiacol was monitored without the addition of benzoquinone or naphthoquinone and shown to be negligible up to 48 h. For select experiments, guaiacol and benzoquinone were further purified from the commercial samples (Supporting Information).

## Results and Discussion

### Enhanced Light Absorption

Since our initial focus was the low-polarity, hydrocarbon-like organic phase of BBOA, we began by exploring the absorption of guaiacol, benzoquinone, and their mixtures in nonpolar heptane. Heptane is a good proxy of the hydrocarbon-like organic phase of BBOA, with an oxygen-to-carbon ratio of zero, like squalane,^31^ a commonly used proxy of primary organic aerosol.^42,43^ Guaiacol and its derivatives, including coniferaldehyde,^44^ are some of the most abundant emissions from the smoldering of conifers,^3,4^ including eastern red cedar,^45^ a species associated with elevated wildfire risk in the southern Great Plains.^46,47^ For example, emission factors for guaiacol and coniferaldehyde are 280 and 230 mg kg^–1^ of pine, respectively.^3^ Values of 21 and 340 have been reported for the 1-octanol–water partitioning coefficient of neutral guaiacol at 298 K,^48^ indicating a moderate to high preference for the low-polarity, hydrocarbon-like organic phase over the aqueous phase, although its precise distribution across the air, water, and organic phases will depend on additional factors (e.g., temperature, pH, and mass and liquid water content of organic aerosol).^49−51^ The 1-octanol–water partitioning coefficient of guaiacol was estimated to be 57 at 298 K below pH 7, using SPARC Performs Automated Reasoning in Chemistry (see Figure S2),^52−54^ and this value lies between the previously reported values,^48^ consistent with a moderate preference for the hydrophobic phase of BBOA. Although biomass burning is also a source of inorganic species, including potassium salts like KCl, KNO_3_, and K_2_SO_4_,^55,56^ we do not consider these in this work because they would be present in the polar, hydrophilic phase rather than the nonpolar, hydrophobic phase.

**Figure 3:**
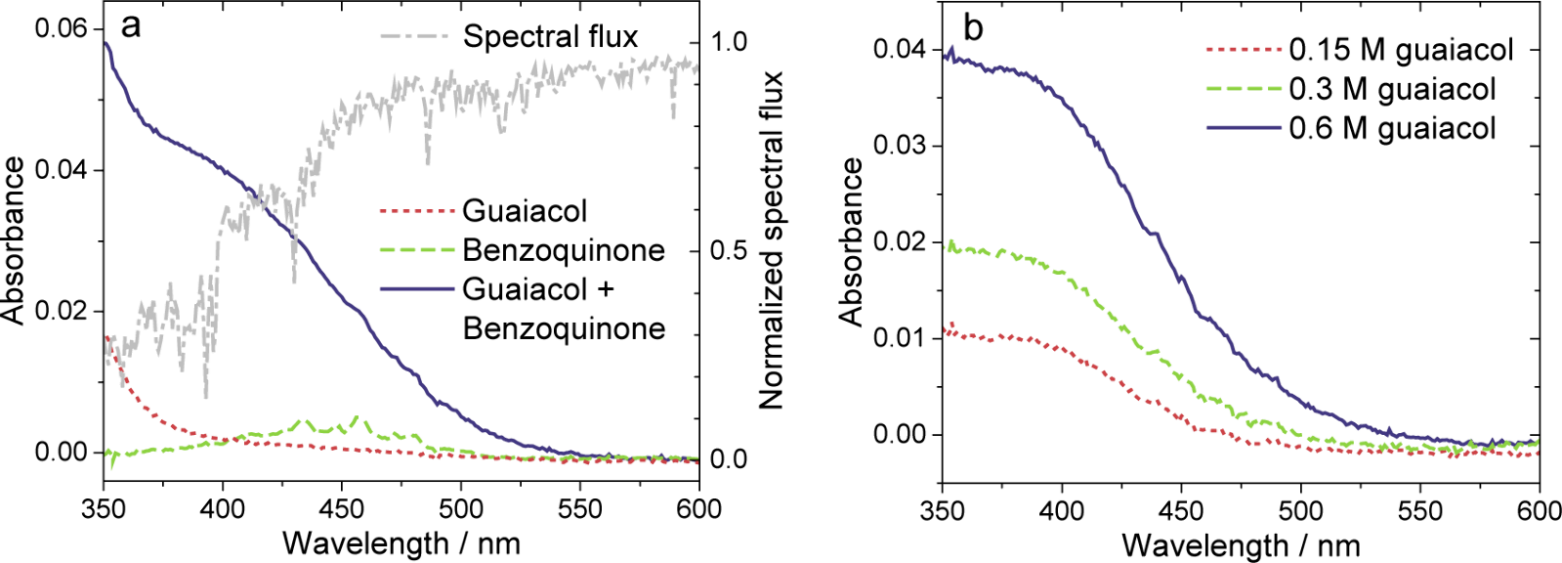
Absorption spectra of (a) solutions of 0.6 M guaiacol, 3.4 × 10^–4^ M benzoquinone, and their mixture at the same concentrations in heptane in a 1 cm cuvette and difference spectra at (b) varying concentrations of guaiacol and 3.4 × 10^–4^ M benzoquinone. The dashed line shows the normalized spectral flux. The spectral flux was calculated as the 24 h average for July 20, 2021, and normalized to that at 700 nm.

Guaiacol is colorless, exhibiting negligible absorption at 380 nm and all visible wavelengths, even at concentrations as high as 0.6 M, as shown in Figure 3a. The absorption of guaiacol at wavelengths below 380 nm has been investigated precisely in the gas phase, using high-resolution spectroscopy.^57^ Benzoquinone exhibits an absorption feature centered at 445 nm, appearing faintly yellow at a concentration of 3.4 × 10^–4^ M. Benzoquinone is much less soluble than guaiacol in heptane; therefore, this concentration is already near the solubility limit. The absorption of benzoquinone at visible and ultraviolet wavelengths has been studied thoroughly in the gas, solution, and crystalline phases,^58−60^ and the weak feature centered at 445 nm is due to excitation from nonbonding molecular orbitals that are mostly composed of *p* orbitals of the oxygen atoms to an antibonding π orbital (i.e., *n*–π* transition).^60^ Upon mixing guaiacol and benzoquinone at concentrations of 0.6 and 3.4 × 10^–4^ M, respectively, in a cuvette, we observed greater absorption at visible wavelengths than for either component individually (see Figure 3a). Subtraction of the spectra of the individual components from that of the mixture highlights this additional absorption. As shown in Figure 3b, this absorption band is centered at about 400 nm and extends beyond 500 nm, such that there is significant overlap with the solar spectral flux at the surface (see Figure 3a), calculated using the Quick Tropospheric Ultraviolet Visible Calculator,^61^ as described previously.^62^ This additional absorption appears instantaneously upon mixing, and it does not increase with time after mixing, as shown in Figure S3. Instantaneous coloration is a general feature of charge-transfer complexes, whereas increasing coloration with reaction time would be expected for the formation of a covalent product of guaiacol and benzoquinone. Furthermore, this additional absorption is concentration dependent, i.e., increasing with the concentration of guaiacol (see Figure 3b). Analogous results were observed for guaiacol and naphthoquinone as well, indicating that this enhanced absorption can occur for electron acceptors besides benzoquinone.

### Temperature Dependence

**Figure 4:**
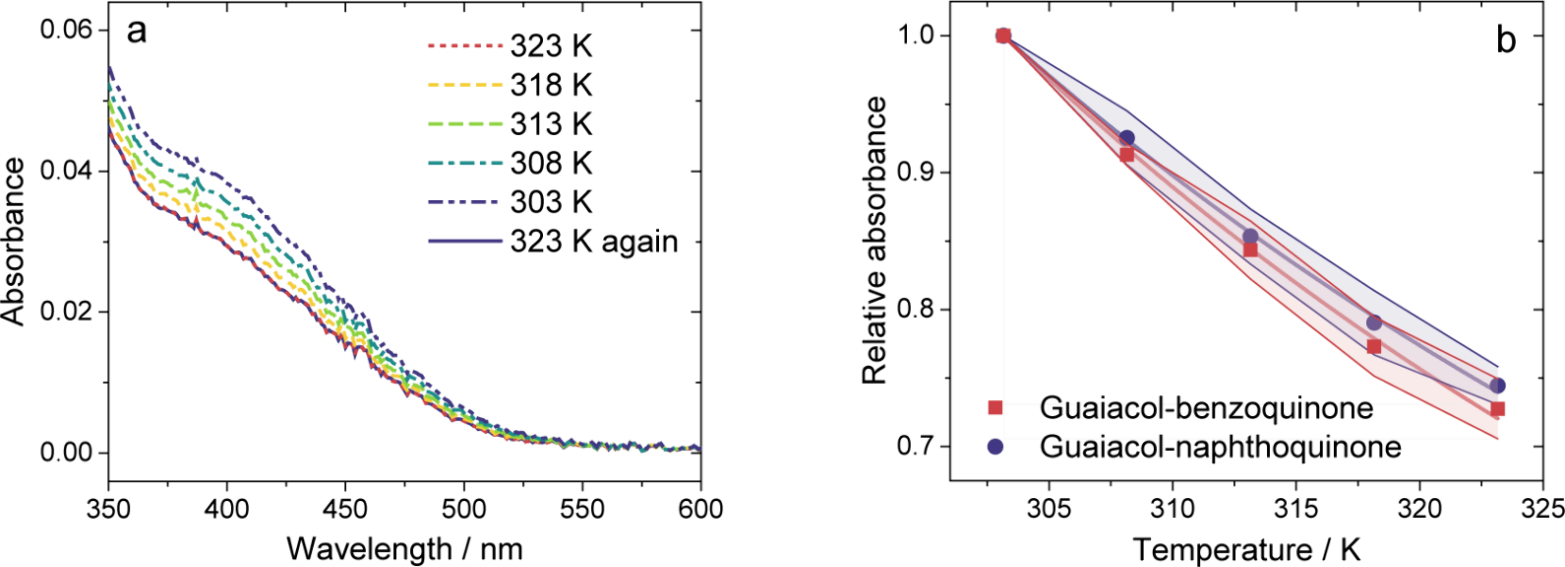
Effect of temperature. (a) Absorbance and (b) relative absorbance at 405 nm of solutions of guaiacol and either benzoquinone or naphthoquinone in heptane in a 1 cm cuvette as a function of sample temperature, after accounting for the absorbance of the quinone. Panel (a) shows a representative experiment with 0.6 M guaiacol and 3.4 × 10^–4^ M benzoquinone. In panel (b), for guaiacol, a concentration of 0.6 M was used; for each quinone, concentrations of 3.4 × 10^–4^ and 6.8 × 10^–4^ M were used. No differences due to concentration were observed, so both conditions were included in the averages. The shaded regions indicate one standard deviation among six replicate experiments. The curves are the fits to the experimental data. Uncertainties in the resulting enthalpies were determined from the 95% confidence intervals of the fits.

If the enhanced absorption originates from noncovalent charge-transfer complexes, it should exhibit temperature dependence. Since intermolecular complexation brings two species, with their own translational degrees of freedom in solution, together into one, the entropy of complexation is always negative, disfavoring spontaneity. Consequently, whenever complexation is spontaneous, it is enthalpy-driven, meaning enthalpy is negative.^14,21^ According to the van’t Hoff equation, then, increasing temperature should shift equilibrium toward the unbound donor and acceptor species, and the initially enhanced absorption should be diminished. Absorbance is directly proportional to the molar absorptivity, ε_DA_, and concentration of the complex, [DA], according to Beer’s law, *A* = ε_DA_*b*[DA], where *b* is the path length. The concentration of the complex can be expressed in terms of the equilibrium constant, *K*:
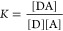
The fractional absorbance at temperature *T* relative to the absorbance at the lowest temperature *T*_0_, *A*(*T*)/*A*(*T*_0_), can be expressed using these relationships. Following McKay et al., we assume that the concentrations of donor and acceptor species can be taken as the same at both temperatures,^14^ which is fair when [DA] is much smaller than [D] and [A]. As a result, the fractional absorbance can be written as the following:
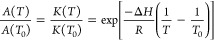
where Δ*H* is the enthalpy of complexation and *R* is the gas constant. The lowest reference temperature, *T*_0_, was taken as 303 K, and *T* was increased in five degree intervals from 303 to 323 K (see Figure 4a). As shown in Figure 4b, across this 20 K interval, the exponential decrease in relative absorbance at 405 nm for the mixture with the highest concentration of guaiacol, 0.6 M, and 3.4 × 10^–4^ M benzoquinone was nearly 25%. Representative spectra are shown in Figure 4a. Temperatures below 303 K were not always accessible at 0.6 M guaiacol because liquid–liquid phase separation occurred, resulting in the formation of colloidal guaiacol and a sudden increase in apparent absorbance, i.e., extinction, due to light scattering. After a sample was left to sit overnight at 277 K in the dark, these colloidal particles settled and formed a highly colored lower layer. The density of guaiacol is 1.11 g cm^–3^, compared to 0.68 g cm^–3^ for heptane. Importantly, all changes observed with temperature were reversible, and returning to the initial temperature at the end of the experiment led to the recovery of the initial absorbance (Figure 4a). Although no temperature dependence of absorbance was previously observed for BBOA extracted into water, the current results are broadly consistent with the slight dependence observed for BBOA extracted into dimethyl sulfoxide.^25^

The enthalpy of complexation, Δ*H*, for guaiacol–benzoquinone can be determined from a fit of the above equation to the experimental data for solutions of guaiacol and benzoquinone. This fit gives a value of −13.3 ± 0.6 kJ mol^–1^ for the enthalpy of complexation. While there are no previous measurements for guaiacol–benzoquinone in any solvent to compare to, we note this result is greater in magnitude than the value measured for the charge-transfer complex between benzoquinone and benzene in heptane, −7.5 kJ mol^–1^.^63^ This difference can be attributed to stronger electrostatic interactions in guaiacol–benzoquinone due to the polar, oxygenated groups of guaiacol, whereas benzene–benzoquinone is more restricted to π–π interactions. Our value for the enthalpy of complexation of guaiacol–naphthoquinone is −12.3 ± 0.4 kJ mol^–1^, based on the results shown in Figure 4b.

### Liquid-Liquid Partitioning

After observing the formation of colloidal guaiacol in heptane and a darkened layer after settling at reduced temperatures in the spectrometer, as described above, we next purposely probed the liquid–liquid partitioning of benzoquinone from heptane to guaiacol. The concentration of guaiacol used above, 0.6 M, was optimized to be close to its solubility limit in heptane, and higher concentrations led to liquid–liquid phase separation, similar to what was observed upon lowering the temperature of the spectrometer. Again, the guaiacol formed a thin layer at the bottom of the cuvette, which was instantaneously dark brown upon mixing, consistent with the partitioning of benzoquinone from the heptane to the guaiacol layer. Charge-transfer complexation between guaiacol and benzoquinone in the absence of solvent, i.e., in the bottom layer, is analogous to widely observed solid-state charge-transfer complexes.^64−66^ One distinction is that solid-state charge-transfer complexes are formed by mechanochemistry, e.g., grinding in a mortar and pestle, in part because the solid, crystalline reagents are highly viscous. Here, liquid guaiacol, in contrast, allows for the rapid diffusion of benzoquinone and subsequent complexation. To demonstrate that this enhanced absorption in guaiacol is steady, as expected for charge-transfer complexes, rather than increasing with time, as expected for covalent products, we next added equal volumes of pure guaiacol and 3.4 × 10^–4^ M benzoquinone in heptane to a cuvette, mixed them, and measured the absorption of both layers in increments of 1 h from mixing. As shown in Figure S4, the enhanced light absorption in the guaiacol layer appears suddenly upon mixing but does not increase within 3 h afterward, consistent with charge-transfer complexation. The steady coloration of the lower guaiacol layer is shown in Figure S5. No change in the composition of the guaiacol layer was observed by liquid chromatography (Figure S6), consistent with the conclusion that complexes, rather than covalently bound products, are responsible for the enhanced absorption. There is a long history of observations of organic charge-transfer complexes,^34,35^ but to the best of our knowledge, this is the first study of complexes with guaiacol.

### Gas-Liquid Partitioning.

Quinones are known to form from the oxidation of aromatic precursors in the gas phase (e.g., naphthoquinone forms from OH-initiated oxidation of naphthalene),^40,41^ so we were interested whether gas–liquid, in addition to liquid–liquid, partitioning could facilitate charge-transfer complexation. For these experiments, we constructed a small exposure chamber that was integrated into a fiber optic-based UV–vis spectrometer. To promote a uniform path length for the spectroscopic measurements, the liquid guaiacol was placed between two round 18 mm cover glasses. Solid benzoquinone was placed in a recessed ring below the liquid sample and had to first sublime to allow gas-to-liquid partitioning into guaiacol. Consequently, the concentration of benzoquinone was dictated by the solid vapor pressure, about 0.1 Torr at 293 K. This experimental design of a sandwiched film with a constant concentration of gas-phase species, i.e., benzoquinone, at the edge is similar to that used in the Raman isotope tracer method, in which the substitution of H_2_O in a film of aqueous levoglucosan, for example, with D_2_O is measured as a function of time.^67,68^

**Figure 5:**
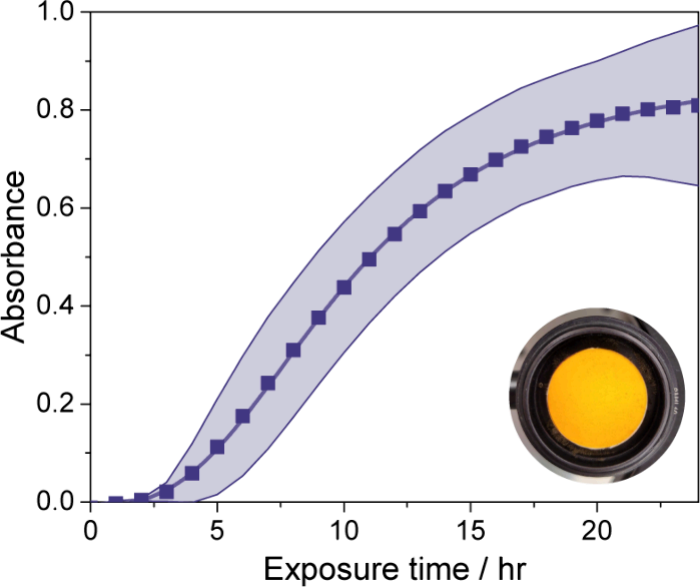
Absorbance at 405 nm of a 20 μm thick film of guaiacol as a function of time, exposed to gas-phase benzoquinone at its solid saturation vapor pressure at 293 K. The shaded region indicates one standard deviation for triplicate experiments. The inset shows the vibrant, uniform color of a thin film against a white background, not present during spectroscopic measurements, after 20 h of exposure to benzoquinone.

Figure 5 shows the evolution of absorbance at 405 nm for a film of guaiacol with a thickness of about 20 μm, associated with a volume of 5 μL, during 24 h of exposure to benzoquinone. The reference spectrum was taken for two cover glasses without guaiacol; therefore, the zero initial absorbance reflects that guaiacol on its own does not absorb light at 405 nm. The film was situated carefully over the observation region of the UV–vis spectrometer, so absorbance measurements are performed across a fixed area distributed about the center of the film. Absorbance began increasing after 3 h of exposure and increased most rapidly between 5 and 10 h. At longer exposure times, the absorbance began to converge and reached a maximum of 0.81 at 24 h. This time dependence is dictated by Fick’s second law,^67^ consistent with the slow diffusion of benzoquinone into the film of liquid guaiacol. The development of color in the film is illustrated in Figure S7. In one experiment, purified starting materials were used, giving the same sigmoidal evolution in color (Figure S8). In the future, it may be valuable to estimate diffusion coefficients for this system using kinetic multilayer modeling,^69,70^ but these simulations are beyond the scope of the current work. Hourly measurements were made for another 24 h after the peak, and a small 15% decrease between 24 and 48 h was observed. This decrease is attributed to the minor loss of guaiacol, which has a vapor pressure of 0.1 Torr at 293 K, from the exposure chamber and, in turn, the film, despite the regular replacement of sealing O-rings. Since this decrease is relatively small, we take the volume of guaiacol and thickness of the film to be constant over the 24 h period shown in Figure 5. We note that the spectral features were the same as those observed for guaiacol–benzoquinone in heptane. Importantly, no changes occurred for films of guaiacol in the absence of benzoquinone. Multiphase experiments were also performed by using naphthoquinone instead of benzoquinone. Since the solid vapor pressure of naphthoquinone is much lower than that of benzoquinone, 0.02 Torr even at the elevated temperature of 323 K, the gas-phase concentration of naphthoquinone at the edge was much lower, and the increase in absorbance was much slower, not reaching a peak even after 48 h. While guaiacol is only one constituent of BBOA, these observations of guaiacol with benzoquinone and naphthoquinone demonstrate that gas-particle partitioning of quinones to BBOA, which is rich in phenolic species, has the potential to enhance its light absorption.

How does this enhanced absorption in guaiacol due to charge-transfer complexation with quinones compare to the light absorption of other types of BrC? To make comparisons, we used the absorbance measurements of the guaiacol films to calculate the absorption coefficient, mass absorption coefficient, and imaginary component of the refractive index. For the film,^71,72^ the extinction coefficient at wavelength λ, α_bulk_^ext^(λ), can be calculated from the absorbance, *A*_10_^film^(λ). The film of pure, nonabsorbing guaiacol at time zero exhibits no extinction, so the extinction coefficient can be equated to the absorption coefficient, α_bulk_^abs^(λ), which can in turn be calculated as follows:
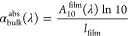
where *l*_film_ is the thickness of the film.^1^ The mass absorption coefficient is calculated from the absorption coefficient and the density of the film, taken as that of guaiacol, 1.11 g cm^–3^, as MAC_bulk_(λ) = α_bulk_^abs^(λ)/ρ_bulk_. We note that MAC_bulk_(λ) does not account for Mie resonances in suspended particles, in contrast to the mass-normalized absorption cross section of the aerosol, MAC_a_(λ).^1,73^ Finally, the imaginary component of the complex refractive index, *k*(λ), can also be calculated from α_bulk_^abs^(λ).^1^
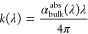
The average value of *A*_10_^film^(λ) at 405 nm at maximum absorption enhancement, after 24 h of exposure to benzoquinone, is 0.81 (see Figure 5). For a film thickness of 20 μm, this value gives an absorption coefficient of 950 cm^–1^, a mass absorption coefficient of 860 cm^2^ g^–1^, and an imaginary component of 0.0031, again all at 405 nm, which is roughly the wavelength at the peak of the new absorption feature (see Figure 3b) due to complexation. The mass absorption coefficient is similar to values reported for secondary organic aerosol from OH-initiated oxidation of naphthalene, about 1000 cm^2^ g^–1^ at 405 nm, which is classified as moderately absorbing BrC.^74^ Secondary organic aerosol from guaiacol also classifies as BrC,^75−79^ but with an imaginary component of about 0.001 at 405 nm,^76^ this is about one-third as absorbing as the complex-containing material reported here. The absorptivity of fresh primary organic aerosol derived from biomass burning is highly variable,^80,81^ but representative *k* values at 405 nm include 0.004 and 0.006 for fuels of ponderosa pine needle litter^82^ and Alaskan duff,^83^ respectively, on the same order as that observed here. The strong wavelength dependence of the enhanced absorption at 400–500 nm (see Figure 3b) results in an absorption Ångström exponent of about 10, which is similar to that recently observed for primary BrC from eastern red cedar.^62^ We qualify this discussion by stating that the optical properties observed here will depend on experimental and environmental factors, including the gas-phase concentration and liquid-phase solubility of the quinone; moreover, the mass absorption coefficient reported here is an effective coefficient for the complex-containing material rather than for the complex itself. Still, these comparisons do serve to show that the complex-containing material here is comparable in mass-normalized absorption to moderately absorbing BrC.

## Atmospheric Implications

These results have implications for the climate impacts of guaiacol and its derivatives,^84^ like coniferaldehyde, which are some of the most abundant phenolic emissions of biomass burning, especially for coniferous (i.e., angiosperm) species.^3,4^ In addition to reacting in the gas phase with atmospheric oxidants,^85,86^ guaiacol also reacts in the particle or aqueous phases to produce light-absorbing secondary organic aerosol or BrC,^75−79,87−90^ including components of nitrophenols,^91−93^ further hydroxylated species,^94,95^ oligomers,^96−98^ and insoluble polyguaiacol inclusions.^99−105^ The products of guaiacol are commonly associated with BrC in field studies,^106−108^ and these include bi- and terphenyl and ether-linked aromatic species. These oligomers typically contain the same functional (i.e., methoxy and hydroxy) groups as the precursor, accommodating the same types of intermolecular interactions; 4,4′-biguaiacol is just one among many such products.^109^ By analogy to increasingly large acceptors that have been shown to complex, from benzoquinone to 3,5-di-*tert*-butyl-1,2-benzoquinone^110^ to naphthoquinone to esculetin,^111^ these increasingly large donors are expected to interact similarly. Indeed, even larger oligomeric donors have been proposed to participate in complexes in Kraft lignin.^110,112^ The SPARC-estimated 1-octanol–water partitioning coefficient of 4,4′-biguaiacol, known to form during atmospheric aging,^109^ is about 6200 at 298 K across all atmospherically relevant pH values (see Figure S2),^113^ indicating that, in addition to having the functionality necessary for the currently reported intermolecular interactions, products of aging can also be distributed in the nonpolar organic phase. Liquid–liquid phase separation of the hydrophobic and hydrophilic phases of BBOA has been observed across all relative humidities at 293 K,^28^ so the occurrence of a nonpolar organic phase is likely widespread. Based on the magnitude of the enthalpies of complexation, the contribution of intermolecular interactions to light absorption will be strongly dependent on atmospheric temperature, and it is expected to increase with altitude since the temperature in the troposphere decreases at a lapse rate of about 10 K km^–1^. As temperature decreases, the partitioning of guaiacol and its derivatives will be shifted to the particle phase, and the particle viscosity will increase, potentially altering uptake, partitioning, and complexation. We note that guaiacol, while an excellent model BBOA component, is just one representative of the substituted phenolic species in biomass burning emissions. In the future, the role of these intermolecular interactions should be investigated for other BBOA components similar to guaiacol and in suspended aerosol particles.

## Supporting Information Available

Materials and methods; figures of SPARC predictions, instantaneous absorption enhancement upon mixing and liquid–liquid partitioning, liquid chromatography, and multiphase results with purified starting materials; and photographs of experimental setup and absorption enhancement upon liquid–liquid and gas–liquid partitioning.(PDF)
