# Supplementary material for: Intermolecular Interactions Enhance the Light Absorption of a Methoxyphenol Constituent of Biomass Burning Emissions
Source: ACS EST Air. 2025 Feb 27;2(3):406–15. doi: 10.1021/acsestair.4c00294 (PMC11915197; doi:10.1021/acsestair.4c00294)
Supplement: Supplementary file 1 — ea4c00294_si_001.pdf [file ea4c00294_si_001.pdf]

Supporting Information for

Intermolecular Interactions Enhance the Light Absorption of a Methoxyphenol Constituent of Biomass Burning Emissions

*Colton T. Calvert, Nathan J. Huskins, and Elijah G. Schnitzler**

Department of Chemistry, Oklahoma State University, Stillwater, OK 74078, USA

E-mail: elijah.schnitzler@okstate.edu.

CONTENTS

SUPPLEMENTARY MATERIALS AND METHODS

Bulk Solution Experiments S3

Multiphase Experiments S3

SUPPLEMENTARY FIGURES

Figure S1. Photograph of chamber for exposure to gas-phase quinones. S5

Figure S2. SPARC predictions for guaiacol and biguaiacol. S6

Figure S3. Instantaneous enhancement in absorbance at 405 nm upon mixing. S7

Figure S4. Instantaneous enhancement in absorbance upon liquid-liquid partitioning. S8

Figure S5. Photograph of absorption enhancement upon liquid-liquid partitioning. S9

Figure S6. Results of liquid chromatography coupled to UV-vis spectroscopy. S10

Figure S7. Photograph of absorption enhancement upon gas-liquid partitioning. S11

Figure S8. Select multiphase experiment with purified starting materials. S12

SUPPLEMENTARY MATERIALS AND METHODS

Bulk Solution Experiments. After mixing in the liquid-liquid partitioning experiment, an aliquot of guaiacol from the lower layer was also analyzed using liquid-chromatography (Agilent, 1260 Infinity) coupled to a diode array detector (Agilent, 1260 DAD HS), in order to monitor for any changes in composition with the occurrence of color. A 20 μL aliquot was diluted in methanol (Fisher Optima, ≥99.9%) and 5 μL of the resulting solution was introduced onto a 2.7 μm C18 column (Agilent, Poroshell 120 EC-C18, 2.1 x 100 mm). The column temperature was 308 K, and the flow rate through the column was 0.3 mL min^{-1}. The mobile phase began as 90% ultrapure water (18.2 MΩ cm), from a commercial water purification system (Thermo, Smart2Pure 3 UV), and 10% methanol (Fisher Optima, ≥99.9%). Formic acid (Fisher Optima) was added to the water to give a concentration of 0.1%. The percentage of methanol was increased linearly to 100% at 20 min and maintained for 5 min. The percentage of methanol was then decreased to its initial value of 10% in 2 min and maintained for 8 min to prepare the column for the next run. The diode array detector was balanced immediately before each run and recorded absorbance at 190-640 nm in steps of 2 nm.

Multiphase Experiments. For select experiments, guaiacol and benzoquinone were further purified from the commercial samples. Benzoquinone was purified by sublimation. The commercial benzoquinone was heated to 353 K during the sublimation. Deposition on glass led to fine, bright yellow crystals. Attempts to crystallize guaiacol from toluene using pentanes did not result in any crystals, even after the mixture sat overnight in a freezer at 253 K. Consequently, guaiacol was purified using preparatory column chromatography, using a gradient of hexane and ethyl acetate. Fractions containing guaiacol were combined, concentrated, and covered in

pentanes to test if crystallization could be induced. Still no crystals formed, so the pentanes were removed under vacuum, along with any remaining volatiles. Both the purified guaiacol and benzoquinone were characterized using ^{1}H NMR, and no impurities were observed.

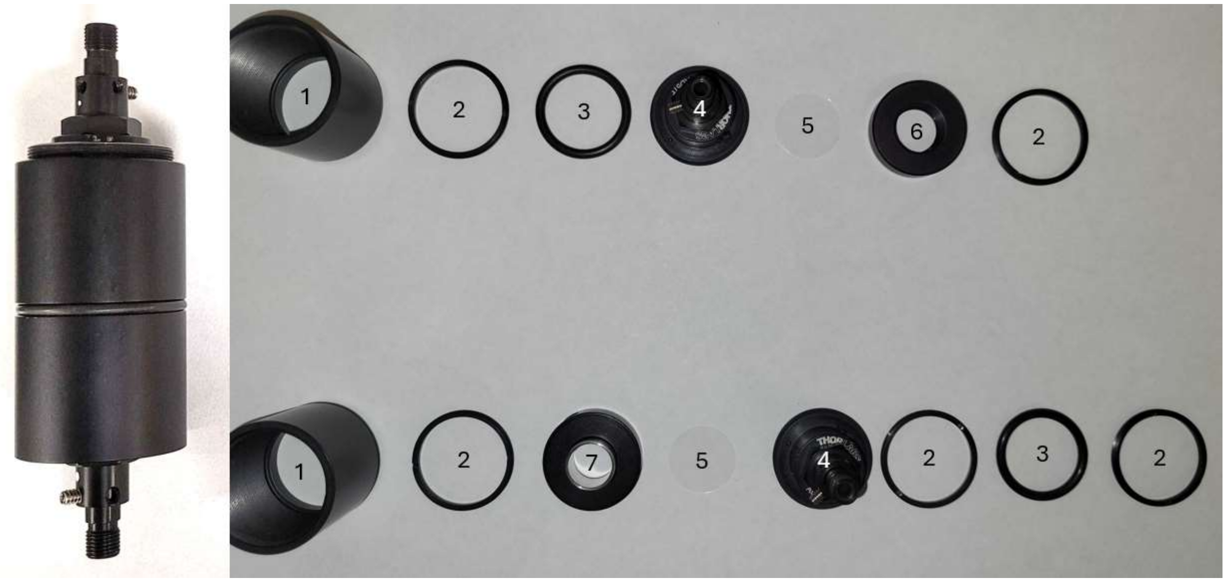

Figure S1. Photographs illustrating the construction of the cylindrical chamber for exposure of thin films to gas-phase quinones and simultaneous UV-vis absorption spectroscopy. The left photograph shows the assembled chamber. The outer walls are those of the lens tubes. The threads at the top and bottom are for fiber-optic connections to the spectrometer and light source, respectively. The right photograph shows the disassembled chamber. Each row shows components for that half of the chamber, in the order they are placed in the lens tube. 1: lens tube (Thorlabs, SM1L10); 2: retaining ring; 3: O-ring; 4: threaded adapter (Thorlabs, AD57F) housing lens (Ocean Insight, 74-UV); 5: cover glass; 6: optic adapter (Thorlabs, AD1T); 7: optic adapter (Thorlabs, AD1) supporting stainless steel spacer tube (Thorlabs, CVH100-COL; note that this model number is for a package of items, one of which is the spacer tube).

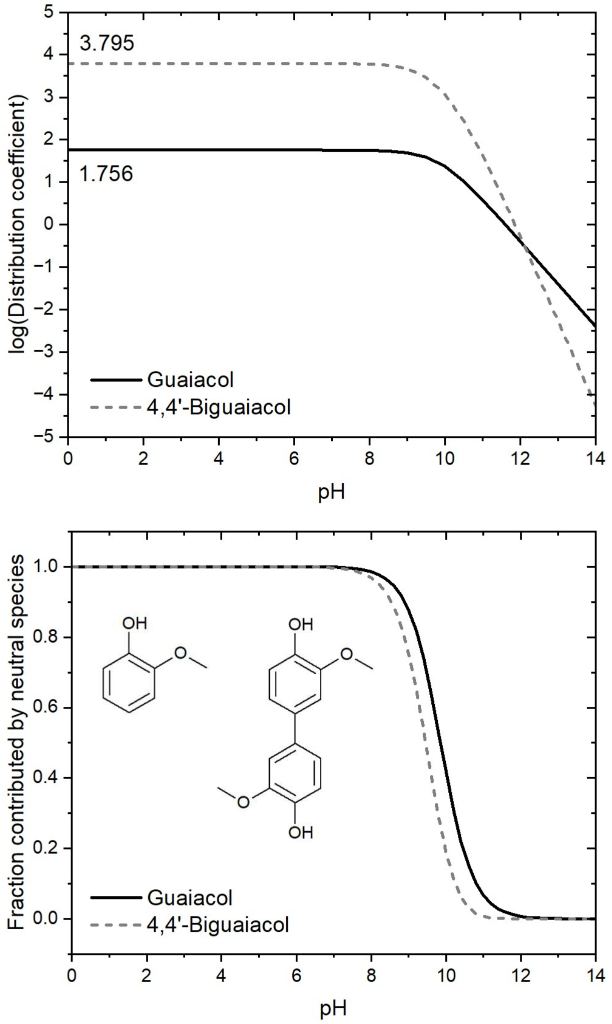

Figure S2. SPARC predictions of the (top) logarithm of the distribution coefficient, log$D$, for all forms of guaiacol and biguaiacol and (bottom) the fraction contributed by the neutral forms of guaiacol and biguaiacol, both as functions of pH. Biguaiacol is a representative product of atmospheric oligomerization of guaiacol, as discussed in the main text. At atmospherically relevant pH values, below 7, both guaiacol and biguaiacol are present in their neutral forms, so the log$D$ values at the left edge of the top figure are the logK_{ow} values for these neutral forms, where K_{ow} is the 1-octanol-water partitioning coefficient.

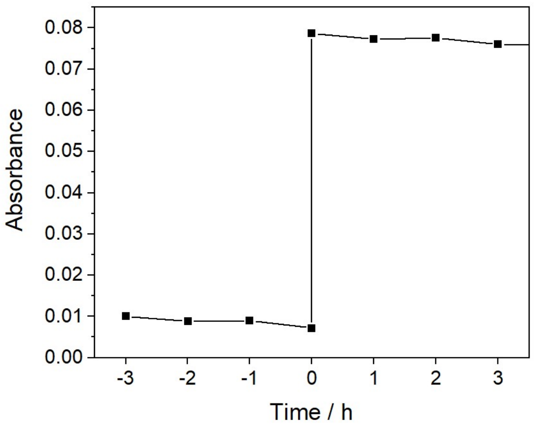

Figure S3. Instantaneous enhancement in absorbance at 405 nm upon mixing guaiacol and benzoquinone in heptane. Before mixing, the absorbance is taken as the sum of guaiacol and benzoquinone individually, and the non-zero values indicate the slight absorbance of benzoquinone at this wavelength.

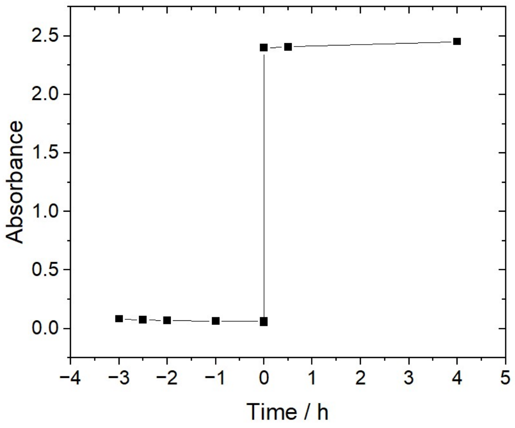

Figure S4. Instantaneous enhancement in absorbance at 405 nm upon liquid-liquid partitioning of benzoquinone from heptane to the liquid guaiacol layer added at time 0 h. The absorbance after partitioning is much higher than that in Figure S3, because the lower layer is pure, rather than dilute, guaiacol.

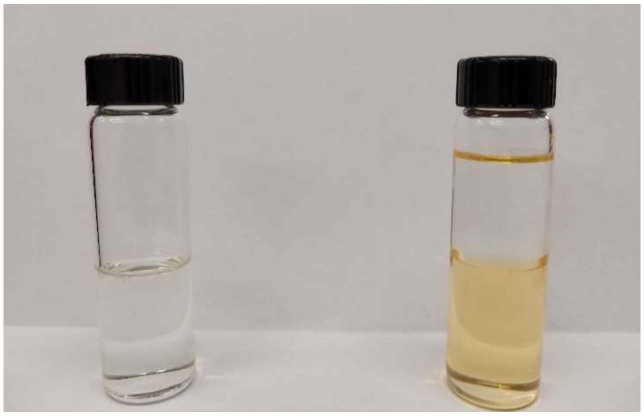

Figure S5. Photograph illustrating the absorption enhancement upon liquid-liquid partitioning. The left vial contains dilute benzoquinone in heptane. The right vial contains the same solution in heptane as the top layer and the added guaiacol as the bottom layer. Alone, guaiacol is colorless, so the yellow color results from liquid-liquid partitioning of benzoquinone from heptane to guaiacol.

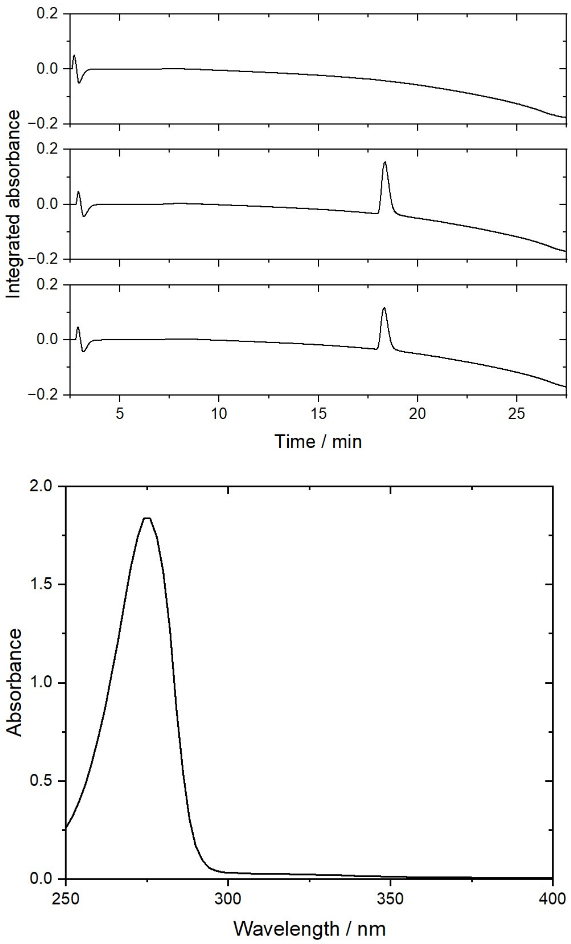

Figure S6. Results of liquid chromatography coupled to UV-vis spectroscopy, using a diode array detector, from the liquid-liquid partitioning experiment. The top panel shows three traces in integrated absorbance from 190 to 640 nm. The first chromatogram is for methanol; the changing baseline is a result of the gradient elution, which changes the absorbance below 250 nm. The second is for pure guaiacol in methanol. The third is for guaiacol sampled from the lower layer in the liquid-liquid partitioning experiment. The peak area for guaiacol is the same, and no new features appear, despite instantaneous coloration. The bottom panel shows the spectrum of guaiacol at the peak in the chromatogram.

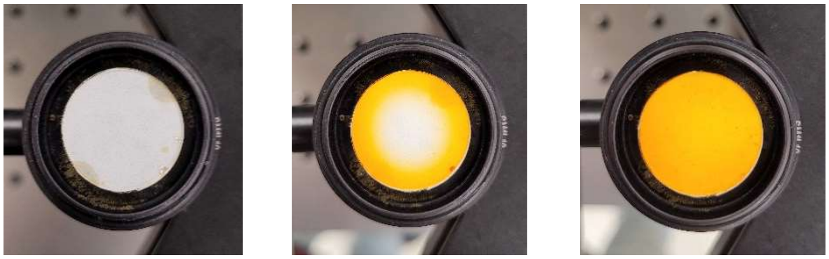

Figure S7. Photographs illustrating the absorption enhancement upon gas-liquid partitioning. The color of the thin films is highlighted by placing the bottom cover glass on a round piece of white paper, which is not present during spectroscopic experiments. The left photograph shows that guaiacol is initially colorless at time 0 h. The center photograph shows a ring of color developing at time 4 h. The right photograph shows uniform, vibrant color across the area of the cover glasses at time 20 h.

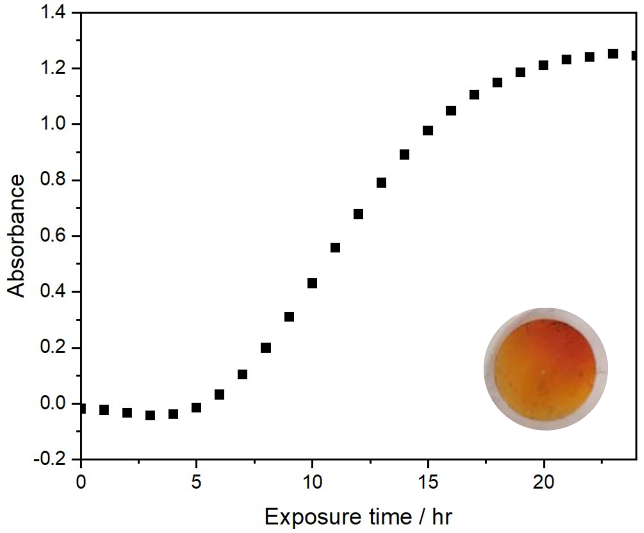

Figure S8. Absorbance at 405 nm of a 20-μm thick film of purified guaiacol supported above purified benzoquinone at 293 K as a function of time. The inset shows the final color of the thin film against a white background.
